# Anti-inflammatory effects of glucagon-like peptide-1 (GLP-1) in coronary artery disease: a comprehensive review

**DOI:** 10.3389/fcvm.2024.1446468

**Published:** 2024-12-16

**Authors:** Alicja Skrobucha, Patryk Pindlowski, Natalia Krajewska, Marcin Grabowski, Szymon Jonik

**Affiliations:** 1st Department of Cardiology, Medical University of Warsaw, Warsaw, Poland

**Keywords:** anti-inflammatory, atherosclerosis, coronary artery disease, glucagon-like peptide-1, mechanisms

## Abstract

Coronary artery disease (CAD)—cardiovascular condition occuring due to atherosclerotic plaque accumulation in the epicardial arteries—is responsible for disabilities of millions of people worldwide and remains the most common single cause of death. Inflammation is the primary pathological mechanism underlying CAD, since is involved in atherosclerotic plaque formation. Glucagon-like peptide-1 (GLP-1) is a peptide hormone which role extends beyond well-known carbohydrates metabolism. In *in vitro* studies GLP-1 receptor agonism is associated with regulation of several inflammatory pathways, including cytokine production, lypotoxicity and macrophages differentiation. In this review, we aimed to provide a comprehensive summary of the potential relationship between anti-inflammatory effects of GLP-1 and CAD. We have described a well-established association of anti-inflammatory properties of GLP-1 and atherosclerosis in animals. Pre-clinical studies showed that anti-atherogenic effect of GLP-1 is independent of modulation of plasma lipid levels and depends on anti-inflammatory response. Human studies in this area are limited by small sample size and often nonrandomized character. However, beneficial impact of GLP-1 on endothelial function and microcirculatory integrity in patients with CAD have been described. Understanding atherosclerosis as a chronic inflammatory disease offers new opportunities for the prevention and treatment of CAD. Therefore, we emphasize the need for larger randomized controlled trials focusing on cardiovascular morbidity and mortality to verify the cardioprotective properties of GLP-1R agonists in patients with CAD.

## 1 Introduction

### 1.1 Atherosclerosis and coronary artery disease

Coronary artery disease (CAD) is a cardiovascular condition occuring due to atherosclerotic plaque accumulation in the epicardial arteries, whether obstructive or non-obstructive (1). The dynamic nature of the CAD process leads to different clinical manifestations, which can be classified as acute coronary syndromes or chronic coronary syndromes (2).

Inflammation plays a key role in the formation of atherosclerotic plaques—the primary pathological mechanism underlying CAD (3). Atherosclerosis as a complex process can be triggered by various cardiovascular risk factors including hypertension, diabetes mellitus, and smoking. The most significant underlying mechanisms include, among others, endothelial dysfunction, increased migration of monocytes, influx of low-density lipoprotein, proliferation of vascular smooth muscle cells and activation of inflammatory molecules (4, 5). Increased oxidative stress can lead to endothelial dysfunction. Activated endothelial cells promote thrombosis, proliferation of smooth muscle cells in vessel wall and increased production of reactive oxygen species, leading to accumulation of macrophages. Plaque formation begins with the accumulation of lipid-laden macrophages. These macrophages absorb oxidized low-density lipoprotein (LDL) particles, and foam cells are formed. Atherosclerotic plaques are abundantly infiltrated with immune cells, leading to an increased expression of inflammatory mediators (6, 7). The subsequent stages of the formation of subendothelial plaque include activation of T cells that release cytokines, growth factors release and activation of smooth muscles, this process leads to the formation of subendothelial plaque. Interleukins (mainly IL-1 and IL-6), interferon-*γ* (IFN-*γ*) and tumour necrosis factor (TNF)-*α*, produced by immune cells, endothelial cells, platelets and fibroblasts are associated with atherosclerosis onset and progression. Over time, the plaque becomes stable, a fibrous cap is formed, and the lesion can become hemodynamically significant resulting in a mismatch between myocardial oxygen demand and supply. This obstruction manifests the symptoms of CAD such as substernal discomfort and a pressure-like feeling, which may radiate to the jaw, shoulder, back, or arm. Some plaques can rupture causing thrombosis, potentially leading to the development of acute coronary syndrome (ACS) (8). CAD remains one of the major cardiovascular diseases affecting people worldwide and accounts for approximately 17.9 million deaths annually (9). Understanding atherosclerosis as a chronic inflammatory disease offers new opportunities for the prevention and treatment of CAD.

### 1.2 Physiology of GLP-1

Glucagon like peptide-1 (GLP-1) is a peptide hormone produced in the intestinal tract by epithelial endocrine L-cells. GLP-1 is produced via the proteolytic cleavage of proglucagon. The main action of GLP-1 is to maintain postprandial glucose homeostasis by enhancing insulin secretion and inhibiting glucagon secretion. It also inhibits gut motility and secretion and regulates food intake. GLP-1 is extremely quickly metabolized and inactivated by the enzyme dipeptidyl peptidase IV (10, 11). The biological effects of GLP-1 are mediated through binding to the specific receptor, GLP-1R, a G-protein coupled receptor that initiates multiple intracellular signaling pathways (12).

### 1.3 GLP-1—diversity of functions

The role of GLP-1 extends beyond carbohydrates metabolism. GLP-1 receptors are expressed in various tissues and organs including liver, kidneys, peripheral and central nervous system, endocrine glands, skeletal muscles and epicardial adipose tissue.

GLP-1 is involved in a complex neuro-hormonal network regulating food intake. GLP-1 regulates skeletal muscle blood flow by recruiting skeletal and cardiac muscle microvasculature (13–16). GLP-1 receptors are expressed in the proximal tubule of the nephron and inhibit reabsorption of tubular fluid, in this way affecting renal hemodynamic and salt handling (17, 18). These receptors are also widely expressed on neurons in the central nervous system, including hippocampal area important for adult neurogenesis and maintenance of cognition and memory formation (19, 20). Previous studies have shown that GLP-1 receptors are expressed in EAT (epicardial adipose tissue) - critically involved in the development and progression of CAD, atrial fibrillation, and heart failure—and are directly correlated with genes promoting FFA (free fatty acid) oxidation and inversely with pro-adipogenic genes (21, 22).

### 1.4 Anti-inflammatory effects of GLP-1

GLP-1 exhibits its anti-inflammatory actions through direct and indirect mechanisms. GLP-1 acts indirectly by improving glycaemic control and weight loss. Beyond its metabolic effects, GLP-1 exerts anti-inflammatory effects via several molecular pathways. In *in vitro* studies GLP-1R- agonists have been found to modulate immune responses. GLP-1R-agonists regulate several inflammatory pathways, including cytokine production, oxidative stress, glucotoxicity, lypotoxicity and the recruitment of immune cells in several organs. GLP-1 acts through its receptors located on immune cells, reducing the production of inflammatory cytokines and infiltration of immune cells in the tissues. In human monocytes GLP-1 is strongly detectable, with waning after differentiation into macrophages (7, 23).

**Figure 1:**
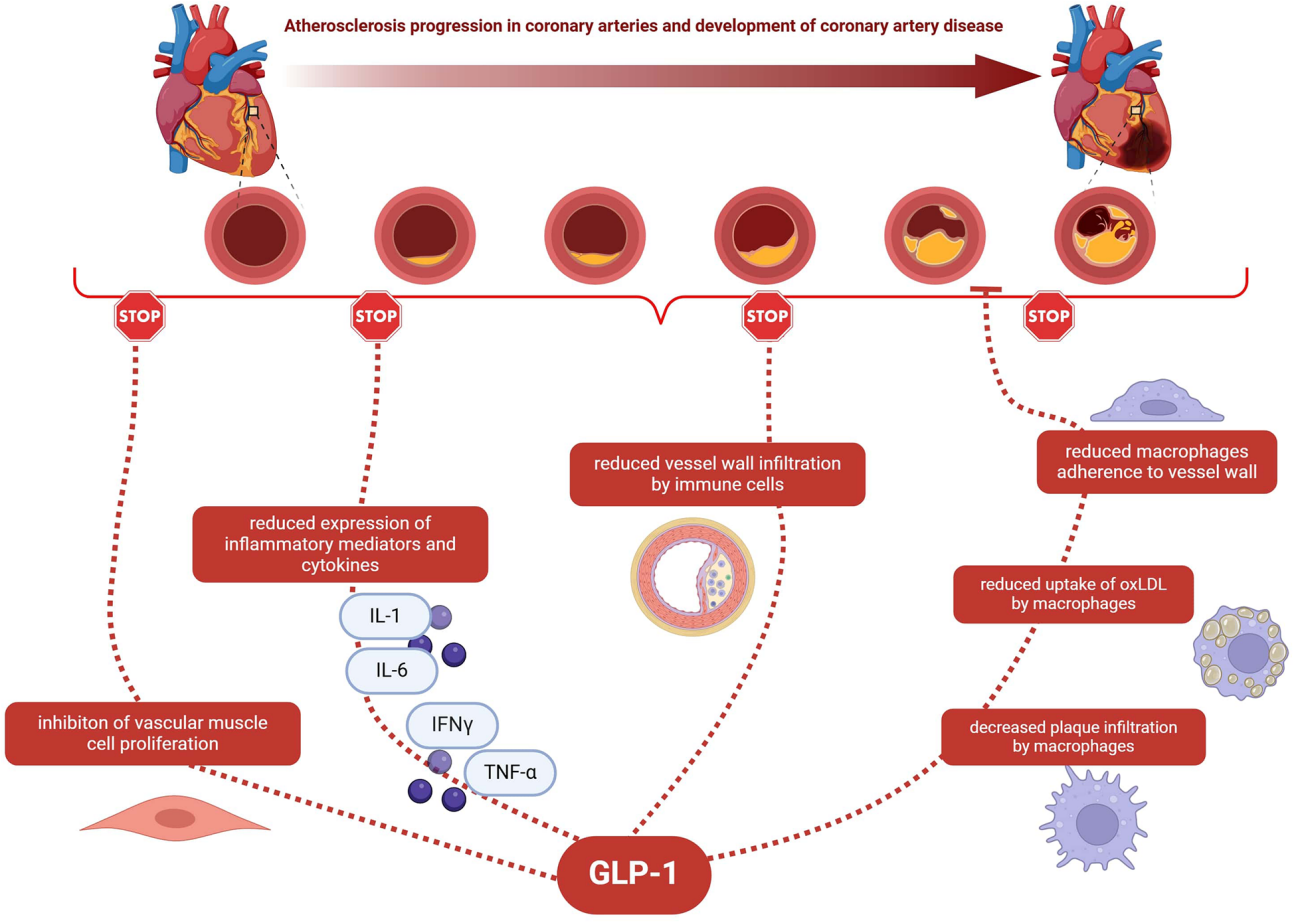
The potential relationship between anti-inflammatory effects of GLP-1 and CAD. GLP-1—glucagon-like peptide-1; CAD—coronary artery disease; IL-1—interleukin 1; IL-6—interleukin 6; IFN*γ*—interferon gamma; TNF-α- *tumour necrosis factor*; oxLDL—oxidized low-density lipoprotein.

The potential relationship between anti-inflammatory effects of GLP-1 and CAD is shown in Figure 1.

## 2 Anti-inflammatory effects of GLP-1 in CAD—insights from animal studies

### 2.1 Anti-inflammatory effect of GLP-1 in atherosclerosis

In a study done by Arakawa M, et al. apoE^−/−^ mice were treated with low- or high dose exendin-4—GLP-1 receptor agonist (24). This resulted in lower adherence of monocytes to endothelial cells of the thoracic aorta compared to the control group by downregulating adhesion molecules such as ICAM-1 and VCAM-1. Furthermore, GLP-1 receptors were found to be amply expressed in monocytes/macrophages. Hence, there was a suspicion of exendin-4's involvement in the inflammatory process and, indirectly and directly, in atherosclerosis development, which was investigated and then validated. Exendin-4 had anti-inflammatory properties by activating adenylate cyclase (cAMP) in macrophages, which inhibited the expression of TNF-α (tumor necrosis factor *α*) and MCP-1—inflammatory and atherogenic mediators. Both TNF- *α* and MCP-1 promote evolution of atherosclerotic lesions and consequently their deficiency reduce those lesions. In summary, Arakawa M, et al. proved GLP-1 receptor activation by inducing the cAMP/PKA pathway in macrophages inhibits atherosclerosis through regulation of inflammatory process. This might have a bearing on the CAD development and its course.

Study done by van Eenige R, et al. on female APOE*3-Leiden. CETP mice injected with either vehicle, a GIPR agonist (GIPFA-085), a GLP1R agonist (GLP-140) or both agonists presented similar results (25). GIPR/GLP1R agonism along with individual GIPR agonism or GLP1R agonism reduced the expression of TNF- *α* and proatherogenic peptides and systemic inflammation markers ICAM-1 and VCAM-1 involved in monocytes/macrophages adhesion to endothelium. Additionally, monocyte and macrophage marker cluster of differentiation 68 (Cd68) and monocyte chemoattractant protein-1 (Mcp-1) levels were lowered when treated with GIPR/GLP1R agonists. The same observation was made in regard to scavenger receptor CD36 expressed by circulating monocytes. CD36 is considered a biomarker for atherosclerotic lesion progression since it stimulates inflammatory response and foam cell formation. All above is consistent with anti-inflammatory effect of GIPR/GLP1R agonism, which plays a role in improving atherosclerosis lesion severity.

Anti-inflammatory effects of GLP-1 on atherosclerotic plaques were also demonstrated by Burgmaier M, et al. (26). Split products of GLP-1, GLP-1(7–37), GLP-1(9–37) and GLP-1(28–37), as well as LacZ (control) carried in AVV vector system were injected into apoe^−^/^−^ mice on high-fat diet. They remained on the vector injection for total of 12 weeks. Atherosclerotic plaques' morphology and stability in the descending aorta of mice were assessed. No changes in the atherosclerotic lesions size were detected, however, plaque vulnerability was reduced. GLP-1 and its split products [GLP- 1(9–37) and GLP-1(28–37)] limited plaque macrophage infiltration and MMP-9 expression. MMP-9 expression is up-regulated by macrophages and is responsible for degrading extracellular matrix proteins including collagen, which results in thinning of the plaque's fibrous cap. Hence, inhibition of MMP-9 secretion by GLP-1 and its products results in thickening of the fibrous cap and increased collagen content of the plaque, improving its stability. This might be clinically useful in patients with type 2 diabetes, who are more susceptible to inflammation and rupture of atherosclerotic lesions.

By gene profiling of aorta samples and evaluation of anti-inflammatory properties of semaglutide in an acute *in vivo* inflammation model Rakipovski G, et al. (27) showed GLP-1RAs (glucagon-like peptide-1 receptor agonists) take part in modulation of atherosclerosis. LDLr^−/−^ and ApoE^−/−^ mice on a Western diet (WD) were given daily injections of liraglutide or semaglutide or vehicle control for 12 to 17 weeks (12 to 14 weeks in ApoE^−/−^ mice and 17 weeks in LDLr^−/−^ mice). Gene expression of inflammatory markers associated with leucocyte recruitment, adhesion, and migration in atherosclerotic aortas was evaluated. It was reduced by semaglutide, which prompted further evaluation of anti-inflammatory properties of semaglutide in an acute *in vivo* inflammation model. Lean C57BL/6J mice were injected with semaglutide and then LPS. By measuring TNF- *α* and IFN-*γ* levels, inflammatory cytokines responsible for systemic inflammatory response, semaglutide was proved to lower them, thus, reduced the systemic inflammation. Those findings might explain the reduction of aortic plaques areas independent of body weight changes by semaglutide and liraglutide in this study. This led to conviction that GLP-1RAs play a role in the protection against atherosclerosis, mediated by reduction in inflammatory pathways.

Semaglutide role in vascular inflammation was also investigated by Jensen J.K, et al. (28). A total of 23 atherosclerotic New Zealand White rabbits were randomized to receive semaglutide or saline-placebo treatment. After 16 weeks PET/CT scan was done with the use of different radiotracers: one for imaging of activated macrophages, one for imaging cellular metabolism and one for visualizing micro-calcifications. Rabbits receiving semaglutide had a lower uptake of tracers imaging activated macrophages and cellular metabolism, but no difference with placebo group in imaging vascular microcalcification was observed. This demonstrates anti-inflammatory role of long acting GLP-1RA in atherosclerosis through decreased activity of macrophages.

Another study investigated the influence and underlying mechanism of exendin-4, a glucagon-like peptide 1 receptor agonist, on inflammation in both liver and vessel walls simultaneously. Wang Y, et al. fed female APOE*3-Leiden.CETP mice cholesterol-containing western-type diet for 5 weeks to induce atherosclerosis and then treated them with exendin-4 for the next 4 weeks (29). Exendin-4 decreased atherosclerotic severity and area by almost 33%. This was explained by reduction of monocyte adhesion to the vessel wall and macrophage content in the plaque. Additionally, exendin-4 reduced the macrophages uptake of oxLDL by activating GLP-1 receptor and as a consequence, reduced foam cell formation *in vivo*. Since treatment with exendin-4 only modestly lowered cholesterol level and affected lipoprotein profile of the mice, anti-atherogenic effect was independent of modulation of plasma lipid levels and dependent on anti-inflammatory response.

Suppressed macrophage infiltration followed by reduced macrophage foam cell formation by active forms of GLP-1 and GIP was demonstrated by Nagashima M, et al. (30). This proved an anti-atherosclerotic effect of GLP-1 and GIP. In this study, ApoE^−/−^ mice were treated with GLP-1(7–36)amide, GLP-1(9–36)amide, GIP(1–42) or GIP(3–42) injections for the course of 4 weeks. Afterwards, variables such as atherosclerosis, oxidised LDL-induced foam cell formation and related gene expression in exudate peritoneal macrophages were assessed. Both GLP-1(7–36)amide and GIP(1–42), active forms of GLP-1 and GIP, significantly reduced the atherosclerotic lesion area and plaque size, whereas GIP(3–42) and GLP-1(9–36)amide did not present the same effect. The anti-atherosclerotic effect of active forms was cancelled by exendin(9–39) or Pro3(GIP)—specific antagonists for GLP-1 and GIP receptors. Moreover, GLP-1(7–36)amide and GIP(1–42) decreased oxidised LDL-induced cholesteryl ester accumulation, CD36 and ACAT-1 (acyl-coenzyme A:cholesterol acyltransferase-1) protein levels in macrophages. CD36 and ACAT-1 are connected with macrophage foam cell formation and aortic smooth muscle cell proliferation. Another pathway through which macrophage cell formation was suppressed was via cAMP activation. GLP-1 and GIP were also responsible for down-regulating the expression of MCP1, VCAM1, ICAM1 and PAI1—molecules regulating inflammatory response.

Similar results were obtained by Tashiro Y, et al. (31). Anti-atherogenic effects of GLP-1, GIP, exendin-4 in primary cultured human monocyte-derived macrophages and liraglutide in apoE^−/−^ mice were studied. GLP-1(7–36)amide, GIP(1–42), and exendin-4, or liraglutide, and a low dose of vidagliptin (to prevent incretins degradation) were added to the human peripheral mononuclear cells cultures and incubated for a week. On the other hand, liraglutide or saline were infused into apoE^−/−^ mice for total of 4 weeks. The effects of both constituents were as follows. GLP-1, GIP, exendin-4 and liraglutide had suppressive effects on oxLDL-induced foam cell formation, which is linked with the suppression of infiltrated macrophage foam cells in atherosclerotic plaques, resulting in repressed atherosclerotic plaque formation. The underlying mechanism of this phenomenon was down-regulation of ACAT1 and CD36 and ABCA1 up-regulation. Liraglutide in apoE^−/−^ mice reduced body weight as well as suppressed macrophage-driven atherosclerosis. It reduced the surface area of the lesions, lessened the plaque size and monocyte/macrophage accumulation. Additionally, as in cell cultures, liraglutide suppressed CD36 and ACAT1 expression in macrophages but did not affect ABCA1 expression. In conclusion, incretins and GLP-1 analogs by suppressing macrophage cell formation prevent the development of atherosclerotic lesions.

The same study construct was used in a study done by Bruen R, et al. (32). Liraglutide effect on atherosclerosis was studied on both human atherosclerotic plaque cultures and human peripheral blood macrophages as well as apoE^−/−^ mice on a high-fat, high-cholesterol diet. GLP-1 receptor agonist inhibited proinflammatory monocyte chemoattractant protein-1 secretion from human atherosclerotic plaque samples and monocyte chemoattractant protein-1 (MCP-1), tumor necrosis factor-α (TNF-α), and interleukin (IL)-1β secretion from human macrophages after ex vivo treatment. Additionally, liraglutide increased IL-10 secretion and CD206 gene expression in human macrophages, which also promoted anti-inflammatory effect. Study on apoE^−/−^ mice proved that liraglutide reduced atherosclerosis. Analysis of the mice' bone marrow cells, precursors of the monocytes, showed upregulation of proinflammatory cathepsin protein family (CatB and CatZ), which was later terminated during bone marrow differentiation to the macrophages. That was further proved in the flow cytometry of the differentiated bone marrow. It showed lowered proinflammatory (M1) and increased anti-inflammatory (M2) macrophages—no increase in CatB or CatZ expression was observed. Those findings were consistent with a different research (33) that showed proinflammatory monocytes were required for regression of atherosclerosis, which would suggest that increase of proinflammatory proteins such as CatB and CatZ in monocyte precursor cells may be associated with the inhibition of atherosclerosis. Subsequent differentiation to M2 macrophages, under the influence of liraglutide treatment, also proved the anti-atherosclerotic effect of GLP-1 receptor agonist since M2 macrophages have been numerously identified in murine atherosclerosis regression models.

Macrophages phenotype dictated by liraglutide and its effect on atherosclerosis was again researched by Bruen R, et al. (34). Inflammatory response to liraglutide was investigated on human THP-1 macrophages (later polarized into M0,M1,M2 macrophages) and bone marrow-derived macrophages, from both wild-type C57BL/6 (WT) and apolipoprotein E null mice (apoE−/−). Additionally, atherosclerotic lesions in aortae from apoE−/− mice were analysed. Liraglutide by promoting anti-inflammatory monocyte/macrophage populations and reducing pro-inflammatory populations decreased atherosclerotic lesion formation. TNF-α and MCP-1 expression was once again attenuated in human peripheral blood mononuclear cells. Liraglutide also decreased IL-1beta in M0 THP-1 macrophages and bone marrow-derived macrophages from WT mice and induced a significant increase in the M2 surface marker mannose receptor in both M0 and M2 macrophages. This hinted on liraglutide being an immune modulator that can alter macrophage phenotype. Moreover, in *in vivo* setting, liraglutide promoted M2 macrophage phenotype by the expression of M2 macrophage markers, Arg-1 and CD163, which suggested liraglutide inhibition of atherosclerosis development. Besides, M1 macrophage markers expression, CCR7, TNF- *α* an IL-6 was lowered. To further verify those findings, flow cytometry of the bone marrow differentiated cells was performed. There was a decrease in inflammatory monocyte population and tendency for differentiation towards M2 macrophages following liraglutide treatment, which was in line with previous findings. In short, liraglutide, GLP-1 receptor agonist, has a direct effect on atherosclerosis lesion formation via promoting pro-resolving M2 macrophages phenotype.

Direct influence of GLP-1 on CAD has been researched by Bose A.K, et al. (35). GLP-1 with VP (valine pyrrolidide) was given intravenously before induced heart ischaemia of the male Sprague-Dawley rats. The ischaemia-inducing procedure and later reperfusion was done both *in vivo* and *in vitro*. GLP-1 + VP infusion exhibited significant reduction in infarct size in both isolated and intact heart compared to the control group (VP group or saline group). To examine molecular mechanisms of this phenomenon, exendin (9–39)—specific inhibitor of GLP-1 receptor, Rp-cAMP—the cAMP inhibitor, PI3 K inhibitor LY294002 or p44/42 mitogen-activated protein kinase inhibitor UO126, were given concomitantly with GLP-1 + VP infusion. They abolished the myocardial protection properties of GLP-1. This proves that GLP-1 promotes the activity of PI3 K in *β*-cell, which is connected with myocardial protection in ischaemia/reperfusion injury through antiapoptotic effect. Additionally, abolishment of myocardial protection by the PI3 K inhibitor LY294002 and by the p44/42 mitogen-activated protein kinase inhibitor UO126 implicates that both of this prosurvival pathways take part in cardioprotection mediated by GLP-1. Inhibition of GLP-1 by cAMP inhibitor also confirms cAMP pathway role in myocardial protection. This study, for the first time, proved cardioprotective effect of GLP-1 in rat hearts and gave the foundation for exploring new theraputical potential of GLP-1 agonists.

Another study exploring the effect of GLP-1 analogue on infarct size was done by Timmers L, et al. (36). Animals subjected to ischaemic injury and then reperfusion were pigs. Treatment with exenatide (or phosphate-buffered saline as control group) was initiated 5 min before the onset of reperfusion and then continued for 2 days. Additional pigs were included to harvest myocardial tissue to asses molecular pathways involved in cardioprotection. Exenatide caused a 40% reduction of myocardial infarct size and significantly improved systolic and diastolic function. On the molecular level, exenatide promoted the expression of prosurvival kinase pAkt as well as antioxidant enzyme superoxide dismutase and catalase responsible for nuclear oxidative stress reduction but inhibited the expression of caspase 3 that plays a central role in cell apoptosis. This proved GLP-1 analogue, exenatide, to have cardioprotective properties, where one of the underlying mechanisms is oxidative stress reduction.

Study by Diebold S, et al. investigated GLP-1 secretion after myocardial infarction (37). Plasma samples from patients presented with indication of coronary artery were assessed for GLP-1 levels. Endogenous GLP-1 in blood samples from patients presented with STEMI (ST-elevation myocardial infarction) was higher in comparison to patients with angiographically excluded CAD. The second part of this study was conducted on C57BL/6J and GLP-1 receptor KO mice. Mice were pretreated with DPP4 inhibitor linagliptin, Exendin-9, GLP-1 (7–36) or GLP (9–36) 1 day and 1 h prior to the ischaemia-inducing procedure. Circulating GLP-1 concentrations increased after induced myocardial infarction in a murine model. This was most likely caused by induction of IL-6 after MI (significant correlation between IL-6 and GLP-1 plasma levels was observed). Additionally, GLP-1 was responsible for increased left ventricular contractility, independent of glucose metabolism. This was related to GLP-1 effect on AMPK activation with downstream induction of mitochondrial respiratory capacity in non-infarcted areas of the heart.

Vinué et al. demonstrated that the GLP-1 analogue lixisenatide decreases atherosclerosis in insulin-resistant Apoe−/−Irs2+/− mice by promoting macrophage polarization towards an anti-inflammatory M2 phenotype, which was associated with enhanced plaque stability (i.e., thicker fibrous caps, fewer necrotic cores, and decreased inflammatory infiltrates). These findings highlight the critical role of GLP-1 analogues in modulating immune responses within atherosclerotic lesions, supporting their potential to reduce cardiovascular risk beyond their effects on glycaemic control (38). See Table 1.

**Table 1:** Animal studies evaluating the anti-inflammatory effect of GLP-1 in atherosclerosis.

Study type	Clinical characteristics	Conclusions	Ref. no.
Prospective	6-week-old mice (*n* = 19) with apolipoprotein E knockout (ApoE−/−)	– Exendin-4 by activating cAMP in macrophages inhibited the expression of TNF-α and MCP-1, inflammatory and atherogenic mediators.	(24)
Prospective	9 to 14-week-old APOE*3-Leiden.CETP mice (*n* = 128)	– GLP1R agonism as well as GLP1R agonism alone reduced the expression of TNF- α and also ICAM-1 and VCAM-1 involved in monocytes/macrophages adhesion to endothelium – GLP1R attenuates CD68, MCP-1 and CD36 expression	(25)
Prospective	6-week-old mice (*n* = 40) with apolipoprotein E knockout (ApoE−/−)	– GLP-1 and its split products [GLP- 1 (9–37) and GLP-1 (28–37)] limited plaque macrophage infiltration and MMP-9 expression – MMP-9 inhibition results in improved plaque stability	(26)
Prospective	6 to 8-week-old mice (*n* = 126) with LDLr knockout (LDLr-/-) and 7 to 10-week-old mice (*n* = 180) with apolipoprotein E knockout (ApoE−/−)	– Semaglutide reduced gene expression of inflammatory markers associated with leucocyte recruitment (IL-6, IL-1RN, CCL2, adhesion (SELE, VCAM-1), and migration in atherosclerotic aortas. – In *in vivo* inflammation model semaglutide was proved to reduce systemic inflammation (TNF-α and IFN-*γ* levels reduction). – Reduction in aortic plaque area was independent of body weight changes.	(27)
Prospective	11-week-old atherosclerotic New Zealand White rabbits (*n* = 23)	– Rabbits receiving semaglutide had a lower uptake of tracers imaging activated macrophages, which demonstrated anti-inflammatory role of GLP-1RA in atherosclerosis through decreases activity of macrophages	(28)
Prospective	12-week-old *E3l.CETP* transgenic mice (*n* = 34)	– Exendin-4 reduced monocyte adhesion to the vessel wall, macrophage content in the plaque and macrophages uptake of oxLDL – Anti-atherogenic effect was independent of plasma lipid levels and dependent on anti-inflammatory response	(29)
Prospective	17-week-old mice (*n* = 346) with apolipoprotein E knockout (ApoE−/−)	– GLP-1 (7–36)amide decreased atherosclerotic lesion area and plaque size through reducing oxidised LDL-induced cholesteryl ester accumulation, lowering CD36 and ACAT-1 levels in macrophages and cAMP activation. – GLP-1 down-regulated molecules regulating inflammatory response (MCP1, VCAM1, ICAM1 and PAI1)	(30)
Prospective	Human peripheral mononuclear cells from 38 healthy volunteers 17-week-old mice *(n* = 29) with apolipoprotein E knockout (ApoE−/−)	– GLP-1, exendin-4 and liraglutide had suppressive effects on oxLDL-induced foam cell formation by down-regulating ACAT1 and CD36 and up-regulating ABCA1 – Liraglutide suppressed CD36 and ACAT1 expression in macrophages	(31)
Prospective	Human atherosclerotic plaques obtained postendarterectomy and human peripheral blood macrophages 8-week-old C57BL/6J mice with apolipoprotein E knockout (ApoE−/−)	– Liraglutide promotes upregulation of proinflammatory cathepsin protein family in monocytes precursor, which is connected with regression of atherosclerosis – GLP-1RA increases anti-inflammatory macrophages (M2) in the differentiated bone marrow numerously identified in atherosclerosis regression models	(32)
Prospective	8-week-old mice (*n* = 32) with apolipoprotein E knockout (ApoE−/−) Human THP-1 macrophages and bone marrow-derived macrophages, from both wild-type C57BL/6 (WT) and apolipoprotein E null mice (apoE−/−)	– Liraglutide promotes pro-resolving M2 macrophages phenotype in *in vivo* setting by the expression of Arg-1 and CD163 (M2 macrophage markers).	(34)
Prospective	Male Sprague-Dawly rats (*n* = 116)	– GLP-1 reduced the infarct size in both isolated and intact heart. – Cardioprotective properties of GLP-1 were abolished by exendin (9–39), Rp-cAMP, PI3 K inhibitor LY294002 and p44/42 mitogen-activated protein kinase inhibitor UO126, which proved those signaling paths have a role in the myocardial protection.	(35)
Prospective	Dalland Landrace pigs (*n* = 18)	– Exenatide, GLP-1 analogue, reduced the infarct size by 40%. – Exenatide promoted the expression of prosurvival kinase pAkt, antioxidant enzyme superoxide dismutase and catalase and inhibited the expression of caspase 3	(36)
Prospective	Plasma samples from cardiovascular biobank (*n* = 41) 6-week-old male C57BL/6J mice and GLP-1 receptor KO mice	– Myocardial infarction is a stimulus for GLP-1 secretion, probably caused by IL-6 induction after MI – GLP-1 increases left ventricular contractility after MI through AMPK activation	(37)
Prospective	Apoe ^−/−^ Irs2 ^+/−^ mice (*n* = 38)	– Lixisenatide (GLP-1 analogue) significantly reduced atherosclerosis in insulin-resistant mice by shifting macrophage polarization towards an antiinflammatory M2 phenotype.	(38)

## 3 Anti-inflammatory effects of GLP-1 in CAD—insights from human studies

Study performed by Yang, et al. included 49 patients with CAD and 52 cases of health control (HC). They used flow cytometry to investigate the effect of GLP-1/GLP-1R on the polarization of macrophages. In the CAD group, the expression level of GLP-1R_M1 (GLP-1 macrophages M1) was positively correlated with triglycerides (TG) and GLP-1R on the cell membrane of total, M1, and M2 macrophages was lower than that of the HC group. Also, consternation of IL-8 level was significantly lower in CAD group 9).

In 2004, for the first time in human study, Nikolaidis L.A, et al. investigated the safety and efficacy of infusion GLP-1 for Acute Myocardial Infarction (AMI) with Left Ventricular (LV) disfunction. This prospective, single center, nonrandomized pilot study included 10 patients with AMI and LV disfunction after successful primary angioplasty compared with 11 control patients (21 patients in total). Baseline demographic and background therapy were similar and both groups had similar number of patients with diabetes. Plasma GLP-1, glucose, insulin and nonesterified fatty acid (NEFA) levels were measured at baseline and at 24, 48, and 72 h in the GLP-1–treated group. NEFA decreased significantly (478–402 μmol/L (*P* < 0.03) at 72 h. Plasma glucose also tended to decrease during the first 48 h (162–126 mg/dl), accompanied by a parallel decrease in plasma insulin levels (184–118 pmol/L) consistent with the insulinomimetic effects of GLP-1. The enhanced insulin responses and reductions in NEFA were observed without significant hypoglycemia. They shoved that GLP-1 may improve endothelial function and microcirculatory integrity. However, this study was limited by small sample size and nonrandomized character (39).

Another prospective, randomized trial included 58 patients (18 were treated with exenatide) with STEMI and thrombolysis in myocardial infarction TIMI (Thrombolysis in Myocardial Infarction) flow 0. The TIMI flow grading system is a widely used method of grading coronary flow. It was observed that high-sensitivity C-reactive protein (hs-CRP) level decreased significantly over the month and levels of troponin I and creatine kinase-MB were also reduced in the exenatide group. Exenatide may provide cardioprotective effects through several pathways associated with metabolism, contractility, reduction of apoptotic cell death, and anti-inflammatory effect. 1)

Moreover, several large, prospective, randomized studies have been developed to investigate the association between GLP-1 RAs intake in DM 2 patients and a known cardiovascular disease (CVD) or high cardiovascular (CV) risk. As a result, there was a significant reduction in primary endpoints across all studies (CV death, nonfatal MI and nonfatal stroke). In addition, there was a lower incidence of nephropathy and a non-significantly higher incidence of retinopathy (*p* = 0.33) in the liraglutide group in LEADER. 42) In SUSTAIN-6, patients treated with semaglutide had a lower risk of developing or worsening nephropathy, but a higher risk of complications from diabetic retinopathy than those receiving placebo (40). In the dulaglutide in REWIND and albiglutide in HARMONY studies, significant reductions in HbA1c levels were demonstrated. 5).

In subsequent studies: PIONEER-6 and ELIXA the primary endpoints (death from cardiovascular causes, nonfatal myocardial infarction, or nonfatal stroke, hospitalization for unstable angina) were not met in the GLP-1 RAs group, but a significant decrease in glycated hemoglobin was observed. In addition, people taking semaglutide in PIONEER-6 had a moderate reduction in cholesterol levels. 7).

The studies by Anholm C, et al. included 41 patients with newly diagnosed DM2 and high risk of CV events with CAD, showed the effect of the combination of liraglutide and metformin on the lipid subfractions and markers of low-grade inflammation. Tumor necrosis factor alfa (TNF-α) (6.1 (5.3; 7.0) pg/ml was reduced by metformin/placebo −0.2 (−0.6; 0.2) pg/ml (*p* < 0.05) but was not affected by metformin/liraglutide treatment and with no significant difference between treatments. Baseline C-reactive protein (CRP) 2.72 (1.15; 3.85) mg/L was reduced by metformin/liraglutide treatment −0.5 (−1.58; 0.03) mg/L (*p* = 0.01) but not by metformin/placebo, and with no significant difference between treatments. Despite, the combination of liraglutide and metformin resulted in a significant reduction in the low-density lipoprotein 5 (LDL-5) fraction −399.6 (−805.9; 44.9), *p* = 0.01, which is the most atherogenic compared to statins. Researchers showed that the use of GLP-1 inhibitors can be effective in reducing blood vessel inflammation in DM2 and CAD patients by improving the lipid profile and lowering LDL-5 levels. The rate of provision of NEFA to the plasma pool was no significantly reduced by placebo-metformin whereas liraglutide-metformin treatment significantly reduced the rate from 36.6 (10.4) to 25.9 (14.4) μmol/L/min, *p* < 0.001. Liraglutide resulted in a reduction in baseline net rate of lipidoxidation, OXO, of −8.2 (5.1) μmol/L/min *p* < 000.1 compared to placebo. 8).

Another study by Ceriello A, et al. suggest that GLP-1 improves endothelial dysfunction during hyperglycemia and may reduce oxaidative stress generation (41). A control group (*n* = 12) compared to type 2 diabetes mellitus patients (*n* = 16) had lower levels of glycemia, flow-mediated vasodilation (FMD), plasma nitrotyrosine and plasma 8-iso prostaglandin F2*α* (8-iso-PGF2a) after standar meal and oral glucose tolerance test (OGTT) and after two months of insulin treatment. OGTT test after two months of optimized glycemic control shoved that GLP-1 was more effective in lowering: nitrotyrosine 2 h (0.15 ± 0.04 vs. 0.03 ± 0.02, *P* < 0.01), *Δ* 8-iso-PGF2a 2 h (20.3 ± 2.1 vs. 10.2 ± 1.5, *P* < 0.05), *Δ* FMD 2 h (2.1 ± 0.3 vs. 0.3 ± 0.2, FMD, *P* < 0.05) in the study performed with placebo, compared with the previous clamp. The results demonstrated that the infusion of GLP-1 during high level of glycemia significantly protects endothelial dysfunction and decreases hyperglycemia-induced oxidative stress see Table 2.

**Table 2:** Human studies evaluating the anti-inflammatory effect of GLP-1 in atherosclerosis.

Study type	Clinical Characteristics	Conclusions	Ref. No.
Prospective	49 patients with CAD and 52 cases of HC	GLP-1R agonist is independent of the hypoglycemic effect of T2DM and has protective effect on cardiovascular system. In group with CAD level of IL-8 and GLP-1R expression on total macrophages and M2 was lower.	(42)
Prospective	21 patients (42.9% diabetes) with MI and LVEF < 40% after successful PCI (GLP-1 = 10, controls = 11).	Infusion of GLP-1 significantly decrease glycemia, NEFA and may improve endothelial function	(39)
Prospective, randomized	58 patients (25.9% diabetes) who underwent PCI for STEMI (exenatide = 18, controls = 40)	In exenatide group reduction of hs-CRP, CK-MB and TnI was observed	(43)
Prospective, randomized	9,340 DM 2 patients with high risk of CV events (liraglutide = 4,668, placebo = 4,672) from LEADER trial. Median follow-up: 3.8 years	Liraglutide (GLP-1 RA) significantly reduced the incidence of primary endpoint: CV death, nonfatal MI and nonfatal stroke death and overall mortality. There was a lower incidence of nephropathy and a non-significantly higher incidence of retinopathy in the liraglutide group	(44)
Prospective, randomized	3,297 DM 2 patients with high risk of CV events (semaglutide = 1,648, placebo = 1,649) from SUSTAIN-6 trial. Median follow-up: 2.1 years	Semaglutide (GLP-1 RA) significantly reduced the incidence of primary endpoint expanded composite outcome [CV death, nonfatal MI, nonfatal stroke, revascularization (coronary or peripheral), Patients treated with semaglutide had a lower risk of developing or worsening nephropathy, but a higher risk of complications from diabetic retinopathy than those receiving placebo	(40)
Prospective, randomized	9,901 DM 2 patients with either previous CVD or CV risk (dulaglutide = 4,949, placebo = 4,952) from REWIND trial. Median follow-up: 5.4 years	Dulaglutide (GLP-1 RA) significantly reduced the risk of primary endpoint (CV death, nonfatal MI and nonfatal stroke; and nonfatal stroke but not overall mortality, CV death, nonfatal MI, or hospitalization for UA or HF. Significantly reduced HbA1c	(45)
Prospective, randomized	9,463 DM 2 patients with CVD (albiglutide = 4,731, placebo = 4,732) from HARMONY trial. Median follow-up: 1.6 years	Albiglutide (GLP-1 RA) was proved superior to placebo in reduction of primary composite endpoint (CV death, nonfatal MI, and nonfatal stroke; expanded composite outcome (CV death, nonfatal MI, nonfatal stroke, and urgent coronary revascularization for UA; and fatal or nonfatal MI, but not for overall mortality, CV death or stroke (fatal or nonfatal). Significantly reduced HbA1c	(46)
Prospective, randomized	3,183 DM 2 patients with high CV risk (semaglutide = 1,591, placebo = 1,592) from PIONEER-6 trial. Median follow-up: 15.9 months	Semaglutide (GLP-1 RA) was noninferior to placebo for primary composite endpoint CV death, nonfatal MI and nonfatal stroke, overall mortality and components of primary outcome: CV death nonfatal MI and nonfatal stroke. Significant reduction in cholesterol levels and glycated hemoglobin.	(47)
Prospective, randomized	6,068 DM 2 patients with MI or hospitalized for UA within the previous 6 months (lixenatide = 3,034, placebo = 3,034) from ELIXA trial. Median follow-up: 25 months	Lixenatide (GLP-1 RA) was noninferior to placebo in reduction of primary composite endpoint (CV death, nonfatal MI, nonfatal stroke, or hospitalization for UA; HR = 1.02), individual components of primary composite endpoint, and overall mortality (HR = 1.13). The glycated hemoglobin was significantly reduced.	(48)
Prospective, randomized	41 patients (28 with complete data) with CAD and newly diagnosed DM 2	Combination of liraglutide and metformin decreased NEFA level. Liraglutide also reduced the lipidoxidation compared to placebo.	(49)
Prospective, randomised	28 patients (16 type 2 DM, 12 match-healthy control subjects)	Diabetic patients were treated intensively with insulin for 2 months to improve glycemic control and used GLP-1 infusion. In OGTT use of GLP-1 may protect endothelia function during hyperglycemia, reducing oxidative stress generation compared to placebo.	(41)

## 4 Future directions

In this review, we have provided a comprehensive summary of the potential relationship between anti-inflammatory effects of GLP-1 and CAD. We summarized and evaluated evidence regarding GLP-1 from pre-clinical and clinical studies regarding underlying mechanisms of inflammation and atherosclerosis.

The association of GLP-1 with atherosclerosis in animals has been well established, proving anti-inflammatory effects of GLP-1 on atherosclerotic plaques by modulating inflammatory pathways and activity of macrophages. Pre-clinical studies showed that anti-atherogenic effect is independent of modulation of plasma lipid levels and depends on anti-inflammatory response. GLP-1 receptor agonist exerted direct effect on atherosclerosis lesion formation by affecting macrophages phenotype.

Human studies showed a protective role of GLP-1R in the progression of coronary atherosclerosis through several pathways associated not only with metabolism but also with the polarization of macrophages. Studies that have shown the beneficial impact of GLP-1 on endothelial function and microcirculatory integrity in patients with CAD were limited by small sample size and often nonrandomized character. Given the increasing interest in the potential use of incretins in cardiovascular diseases, conducting randomized controlled trials in this direction is warranted. Larger randomized controlled trials with clinical endpoints, focusing on both cardiovascular morbidity and mortality, should be performed to verify the cardioprotective properties of GLP-1R agonists in patients with CAD and select the population in whom it will be most effective.

For the future, we hope GLP-1- based drugs together with antithrombotic medications can be a promising field to improve prevention and management of coronary events, also in nondiabetic patients, resulting in reduced mortality in patients with CAD.
